# The association between the level of specific IgE to molecular components of mites and the expression of CD23 molecule on B lymphocytes in atopic dermatitis patients treated or not treated with dupilumab—Pilot study

**DOI:** 10.1002/clt2.12278

**Published:** 2023-07-22

**Authors:** Jarmila Čelakovská, Eva Čermáková, Petra Boudková, Ctirad Andrýs, Jan Krejsek

**Affiliations:** ^1^ Department of Dermatology and Venereology Faculty Hospital and Medical Faculty of Charles University Hradec Králové Czech Republic; ^2^ Department of Medical Biophysics Medical Faculty of Charles University Hradec Králové Czech Republic; ^3^ Department of Clinical Immunology and Allergy Faculty Hospital and Medical Faculty of Charles University Hradec Králové Czech Republic

## Abstract

**Background:**

The CD23 molecule has an effect on the regulation of IgE synthesis, either by stimulation or inhibition. It is not yet known whether the expression of CD23 on B lymphocytes is related to the level of allergen‐specific IgE antibodies in patients with atopic dermatitis.

**Aim:**

The aim of this pilot study was to evaluate the association between the expression of CD23 molecule on B cells and on their subsets (memory, naive, switched, non‐switched, and total B lymphocytes) and the level of specific IgE to molecular components of mites in atopic dermatitis patients (with and without dupilumab therapy).

**Methods:**

Forty‐five patients suffering from atopic dermatitis were included: 32 patients without dupilumab treatment (10 men, 22 women, average age 35 years), 13 patients with dupilumab treatment (7 men, 6 women, average age 43.4 years) and 30 subjects as a control group (10 men, 20 women, average age 44.7 years). The serum level of the specific IgE was measured using the components resolved diagnostic microarray‐based specific IgE detection assay ALEX2 Allergy Xplorer. In all included patients, the expression of CD23 molecule on B lymphocytes was evaluated with flow cytometry using monoclonal antibodies. For the statistical analysis of the association between expression of CD23 molecule on B lymphocytes and the level of specific IgE to molecular components of mites, we used non‐parametric Kruskal‐Wallis one‐factor analysis of variance with post‐hoc by Dunn's test with Bonferroni modification and the Spearman's rank correlation coefficient; for coefficients higher than 0.41, we report R2 (%, percent of Variation Explained).

**Results:**

The association between the expression of CD23 molecule on B cells and the level of specific IgE to molecular components of mites was confirmed only in patients with dupilumab therapy. In these patients, the highest association was confirmed between the level of specific IgE to Der p 20 and expression of CD23 on switched B lymphocytes (in 48.9%). In patients without dupilumab, the association between the level of specific IgE to molecular components of mites and the expression of CD23 on B cells and on their subsets is low.

**Conclusion:**

Further research is needed to fully understand the underlying mechanism of this phenomenon and its implications for the treatment of atopic dermatitis.

Dear Editor,

The IgE antibody is of great importance in the development of inflammation in allergic diseases. IgE antibody can bind to various cells of the immune system via high‐affinity IgE receptors (FcεRI). Upon binding to this receptor on mast cells and basophils, inflammatory mediators are released and clinical signs of an allergic reaction develop, for example, in the airways or in the skin.^1^ The high‐affinity IgE receptor (FcεRI) on Langerhans cells and dendritic epidermal cells is important for antigen internalization. Antigen presented via IgE‐FcεRI on these cells leads to the expression of the Th2 subset and to allergic inflammation.^1^ In addition to the FcεRI receptor, IgE antibodies can bind to the more distant FcεRII/CD23 receptor. The exact significance of this receptor for IgE antibodies, which is low‐affinity, is not yet fully understood.^1^ This is mainly because the CD23 molecule plays an important role in many immunological processes and, in addition to regulating IgE, is of great importance in the development and growth of normal and leukemic B cells.^2^ The low‐affinity IgE FcεRII receptor CD23 is expressed by activated B cells, monocytes, follicular dendritic cells, eosinophils, and platelets. The CD23 molecule has an effect on the regulation of IgE synthesis, either by stimulation or inhibition. The CD23 molecule is important for the binding of IgE on B lymphocytes, but it is a low‐affinity receptor, where binding of IgE antibodies to this receptor occurs only under certain circumstances, mainly when the IgE antibody is bound in the immunocomplex.^3^, ^4^

It is not yet known whether the expression of CD23 on B lymphocytes is related to the level of allergen‐specific IgE antibodies in patients with atopic dermatitis.^5^ It is also not yet known what role dupilumab may have on this receptor. The effect of dupilumab on IgE antibody production is not fully understood, but it is thought to indirectly affect IgE production by blocking IL‐4 and IL‐13, which affect B lymphocytes.^6^ In our previous study, we evaluated the CD23 expression in B cells in AD patients.^7^ We confirmed significantly higher expression of CD23 molecule on B lymphocytes and their subsets in AD patients with and without dupilumab therapy compared with controls.^7^

The aim of this pilot study was to evaluate the association between the expression of CD23 molecule on B cells and on their subsets (memory, naive, switched, non‐switched, and total B lymphocytes) and the level of specific IgE to molecular components of mites in AD patients (with and without dupilumab therapy).

Forty‐five patients suffering from AD were included: 32 patients without dupilumab treatment (10 men, 22 women, average age 35 years), 13 patients with dupilumab treatment (7 men, 6 women, average age 43.4 years) and 30 subjects as a control group (10 men, 20 women, average age 44.7 years). Patients were selected to match in terms of age, sex and severity of AD before starting biological treatment. Inclusion criteria are 1) age 14 years and over 2) AD as defined by the criteria of Hanifin and Rajka. Patients with moderate and severe forms of AD without dupilumab and patients with dupilumab therapy lasting at least 18 months were included. Exclusion criteria are pregnancy, breastfeeding, and systemic therapy (cyclosporin, systemic corticoids). The serum level of the specific IgE was measured using the components resolved diagnostic microarray‐based specific IgE detection assay ALEX2 Allergy Xplorer. In all included patients, the expression of CD23 molecule on B lymphocytes was evaluated with flow cytometry using monoclonal antibodies. For the statistical analysis of the association between expression of CD23 molecule on B lymphocytes and the level of specific IgE to molecular components of mites, we used non‐parametric Kruskal‐Wallis one‐factor analysis of variance with post‐hoc by Dunn's test with Bonferroni modification and the Spearman's rank correlation coefficient; for coefficients higher than 0.41, we report *R* ^2^ (%, percent of Variation Explained). Our results show, that in patients without dupilumab therapy the highest sensitisation rate was confirmed to molecular components Der p 1 in 15 patients (46.9%), to Der p 23 in 14 patients (43.8%) and to Der f 1 and Der p 2 in 13 patients (40.6%); the highest level of specific IgE was confirmed to molecular component Der f 2 (mean = 45.15 kU_A_/L, 1. and 3. quartile = 36.73, 47.23 kU_A_/L). In patients with dupilumab therapy, the highest sensitisation rate was confirmed to molecular components Der f 1, Der f 2, Der p 7 and Der p 20 in 6 patients (46.2%); the highest level of specific IgE was confirmed to molecular components Der p 2 (mean = 46.53 kU_A_/L, 1. and 3. quartile = 25.6, 47.23 kU_A_/L) and to Der f 2 (mean = 46.53 kU_A_/L, 1. and 3. quartile = 25.6, 47.23 kU_A_/L) (Table 1).

**TABLE 1:** The mean level (1. and 3. quartile) of specific IgE to molecular components of mites in patients with dupilumab (dupilumab +) and without dupilumab therapy (dupilumab −).

	Dupilumab −		Dupilumab +	
Molecular components	Number of patients (% from 32 patients)	The mean level (1. and 3. quartile)(kU_A_/L)	Number of patients (% from 13 patients)	The mean level (1. and 3. quartile)(kU_A_/L)
Der p 1	15 (46.9%)	15.59 (4.67, 32.76)	5 (35.5%)	37.18 (16.59, 74.52)
Der f 1	13 (40.6%)	31.05 (7.82, 43.49)	6 (46.2%)	40.11 (15.66, 56.24)
Der p 2	13 (40.6%)	44.17 (38.19, 46.18)	7 (53.8%)	46.53 (25.6, 47.23)
Der f 2	12 (37.5%)	45.15 (36.73, 47.23)	6 (46.2%)	46.6 (32.04, 50.01)
Der p 5	8 (25.0%)	24.29 (0.86, 39.09)	5 (38.5%)	2.91 (1.23, 37.79)
Der p 7	8 (25.0%)	31.72 (4.66, 44.84)	6 (46.2%)	1.55 (1.2, 4.76)
Der p 10	2 (6.3%)	6.18	0	
Der p 11	2 (6.3%)	2.6	0	
Der p 20	6 (18.8%)	43.96 (1.01, 45.9)	6 (46.2%)	22.63 (12.21, 44.49)
Der p 21	5 (18.8%)	40.89 (22.01, 44.84)	2 (15.4%)	44.19
Der p 23	14 (43.8%)	20.82 (16.24, 44.16)	5 (15.6%)	40.64 (18.09, 45.35)

We show the associations between the expression of CD23 molecule on B cells and the level of specific IgE to molecular components of mites with the Spearman correlation higher than 0.41 (Table 2). This association was confirmed only in patients with dupilumab therapy. In these patients, the highest association was confirmed between the level of specific IgE to Der p 20 and expression of CD23 on switched B lymphocytes (in 48.9%), between the level of specific IgE to Der p 1 and expression of CD23 on memory B lymphocytes (in 37.7%) and between the level of specific IgE to Der p 10 and expression of CD23 on non‐switched B lymphocytes (in 33.3%). In patients without dupilumab, the association between the level of specific IgE to molecular components of mites and the expression of CD23 on B cells and on their subsets is low.

**TABLE 2:** The evaluation of the associations between the expression of activation marker CD23 on B lymphocytes, their subsets and level of specific IgE to molecular components of mites in patients with dupilumab therapy (the Spearman correlation higher than 0.41).

Expression of CD23 on B lymphocytes	Molecular components	Dupilumab no		Dupilumab yes	
		Spearman correlation	% *R* ^2^	Spearman correlation	% *R* ^2^
Expression of CD23 on memory B lymphocytes	Der f 1	0.0553	**3.1**	0.5256	**27.6**
	Der p 1	0.1526	**2.3**	0.6139	**37.7**
	Der p 10	0.2976	**8.9**	0.5154	**26.6**
	Der p 20	0.2909	**8.5**	0.4121	**16.9**
	Der p 21	0.0896	**0.8**	0.4354	**18.9**
Expression of CD23 on naive B lymphocytes	Der p 1	0.1427	**2.0**	0.5106	**26.1**
	Der p 10	0.3108	**9.7**	0.5154	**26.6**
	Der p 11	−0.0738	**0.5**	0.4644	**21.6**
	Der p 23	0.1819	**3.3**	0.4135	**17.1**
Expression of CD23 on switched B lymphocytes	Der f 1	0.0220	**0.1**	0.4181	**17.5**
	Der p 1	0.0790	**0.6**	0.4229	**17.9**
	Der p 10	0.2304	**5.3**	0.5067	**25.7**
	Der p 20	0.2227	**4.9**	0.6989	**48.9**
Expression of CD23 on non‐switched B lymphocytes	Der p 10	0.3751	**14.1**	0.5766	**33.3**
	Der p 20	0.2841	**8.1**	0.4719	**22.3**
	Der p 21	0.1168	**1.3**	0.4980	**24.8**
Expression of CD23 on total B lymphocytes	Der f 1	0.0652	**0.4**	0.4301	**18.5**
	Der p 1	0.1540	**2.4**	0.5450	**29.7**

*Note*: *R* ^2^ in AD patients without dupilumab therapy and in patients with dupilumab therapy is demonstrated. Explanation: *R* ^2^ = percent of Variation Explained, *R* ^2^ of one quantity (dependent) can be explained by another quantity (independent)—the results in percentages explain the association between the monitored parameters.

The results of our study suggest that the more IgE binds to B cells, the less it can sensitize allergic effector cells, such as basophils and mast cells, and induce allergic inflammation. Our results led us to consider the higher frequency of IgE binding to the CD23 molecule on B cells with the origin of IgE‐IC complex with a non‐inflammatory pathway. The results of our study suggest that the IgE‐antigen complex acts in a non‐inflammatory manner, contributing to the anti‐inflammatory effect of dupilumab.

The exact mechanism underlying the association between specific IgE to molecular components of mites and CD23 expression on B cells is not fully understood and requires further research. However, it is hypothesized that dupilumab‐mediated suppression of the IL‐4 signaling pathway may lead to compensatory upregulation of other immune pathways, including the CD23 pathway, as the immune system tries to restore homeostasis. Additionally, it is possible that the reduction of inflammation by dupilumab may lead to increased exposure of B lymphocytes to allergens, which in turn could trigger CD23 expression.

In summary, the higher association between specific IgE to molecular components of mites and expression of CD23 molecule on B lymphocytes in AD patients with dupilumab therapy may be explained by the complex interplay between the immune system, dupilumab ‘s mechanism of action, and exposure to allergens. Further research is needed to fully understand the underlying mechanism of this phenomenon and its implications for the treatment of AD.

To our knowledge, this is the first study evaluating the association between the level of specific IgE to molecular components of mites and CD23 expression on B cells in AD patients with and without dupilumab therapy. We believe that our findings could help to better understand the role of CD23 molecule in B lymphocytes in AD patients with and without dupilumab therapy.

## AUTHOR CONTRIBUTION

Jarmila Čelakovská—the main investigator. Department of Dermatology and Venereology Faculty Hospital and Medical Faculty of Charles University, Hradec Králové, Czech Republic

‐ selection of the patients, dermatological examination and recommendations for the laboratory examination
‐ processing of all results
‐ writing of the manuscript

Eva Čermáková: Department of Medical Biophysics, Medical Faculty of Charles University, Hradec Králové, Czech Republic

‐ statistical analysis

Petra Boudková. Department of Clinical Immunology and Allergy, Faculty Hospital and Medical Faculty of Charles University, Hradec Králové, 50002, Czech Republic

‐ laboratory examination and flow cytometry

Ctirad Andrýs. Department of Clinical Immunology and Allergy, Faculty Hospital and Medical Faculty of Charles University, Hradec Králové, 50002, Czech Republic

‐ control of laboratory examination

Jan Krejsek. Department of Clinical Immunology and Allergy, Faculty Hospital and Medical Faculty of Charles University, Hradec Králové, 50002, Czech Republic

‐ professional cooperation and supervision

## FUNDING INFORMATION

Charles University, Medical Faculty Hradec Králové, Cooperation, INDI 207034 (the financial support was intended for research purposes to examine the immunological profile). The Service Fee was funded by the authors

## CONFLICT OF INTEREST STATEMENT

There is no conflict of interest. Informed consent was obtained from all subjects involved in the study.

## Data Availability

The datasets generated and/or analyzed during the current study are available from the corresponding author on reasonable request.
